# Why did glutamate, GABA, and melatonin become intercellular signalling molecules in plants?

**DOI:** 10.7554/eLife.83361

**Published:** 2023-06-20

**Authors:** Yaron Caspi, Chrysoula K Pantazopoulou, Jeanine J Prompers, Corné MJ Pieterse, Hilleke Hulshoff Pol, Kaisa Kajala

**Affiliations:** 1 Institute of Environmental Biology, Department of Biology, Utrecht University Utrecht Netherlands; 2 Brain Center, University Medical Center Utrecht Utrecht Netherlands; 3 Department of Radiology, Imaging Division, University Medical Center Utrecht Utrecht Netherlands; University of Freiburg Germany; University of Freiburg Germany

**Keywords:** signalling molecules, plant physiology, metabolism, reactive ion species, comparative biology

## Abstract

Intercellular signalling is an indispensable part of multicellular life. Understanding the commonalities and differences in how signalling molecules function in two remote branches of the tree of life may shed light on the reasons these molecules were originally recruited for intercellular signalling. Here we review the plant function of three highly studied animal intercellular signalling molecules, namely glutamate, γ-aminobutyric acid (GABA), and melatonin. By considering both their signalling function in plants and their broader physiological function, we suggest that molecules with an original function as key metabolites or active participants in reactive ion species scavenging have a high chance of becoming intercellular signalling molecules. Naturally, the evolution of machinery to transduce a message across the plasma membrane is necessary. This fact is demonstrated by three other well-studied animal intercellular signalling molecules, namely serotonin, dopamine, and acetylcholine, for which there is currently no evidence that they act as intercellular signalling molecules in plants.

## Introduction

Why do some molecules act as intercellular signalling molecules? One way to answer this question is to study the functions of intercellular signalling molecules from one kingdom in another (Meyerowitz, 2002). Plantae and Animalia are two of the few kingdoms that independently developed multicellularity and the only two that developed extreme multicellularity (Knoll, 2011). Consequently, by studying intercellular signalling in these two kingdoms, one might identify possible evolutionary principles that structure the repertoire of intercellular signalling molecules in multicellular organisms (Gardiner, 2013). In this context, it is interesting to note that several unexpected similarities (but also involving profound differences) between plants and animals do exist on the tissue (Heidstra and Sabatini, 2014), cellular (Wuest et al., 2010) immune system (Jones et al., 2016), proteome (Spitzer et al., 2006), and interactions with microbiota (Stassen et al., 2021) levels.

Here, we looked at the intercellular signalling function in plants of three well-known signalling molecules from animals. These are glutamate, γ-aminobutyric acid (GABA), and melatonin. Both plants and animals use an extensive repertoire of intercellular signalling molecules. In animals, there is some bias toward studying signalling mechanisms acting in the brain, particularly those of neurotransmitters (Deutch, 2013). Due to the wealth of knowledge on the function of these brain-related signalling molecules in animals, it is interesting to look at cases where the same molecules also act as intercellular signalling molecules in plants (however, some molecules of this triad also act in intercellular signalling in animals outside the brain; see Hyland and Cryan, 2010). In principle, any molecule that acts as a signalling molecule in both kingdoms can be used as a test case. Thus, also studying, in animals, highly studied plant intercellular signalling molecules can be used for the same purpose. The main reason to study this specific triad in plants is that while they are highly studied in animals, only recently has convincing evidence accumulated concerning their function as intercellular signalling molecules in plants.

Thus, this review has a double target. On the one hand, we aim to provide the animal-inclined reader with comprehensive data concerning this triad in plants (we assume a basic knowledge of the mode of action of these molecules in animals). On the other hand, the main target of such an exercise is to try to understand the origin of intercellular signalling molecules and the possible reasons they were recruited for the intercellular signalling function. We conclude that it is probably easy to recruit molecules with critical biological functions in other physiological domains such as metabolism and reactive ion species balancing to become intercellular signalling molecules. We also hypothesise that plant–microbe interactions were also a driving force for their adoption into the role of intercellular signalling in plants.

We start with a brief overview of different intercellular signalling modes and provide some criteria concerning what makes a molecule into an intercellular signalling molecule. In doing so, we adopt less common terminology in plant science but do not claim ‘brain-like’ functions or mechanisms in plants. We use this terminology only to form a common basis for inspiring scientists with an animal background to consider these intercellular signalling in a broader context. Next, we discuss in detail the evidence for the function of this triad as intercellular signalling molecules in plants and provide some general review of their additional physiological functions. We also contrast this triad with another triad of well-studied animal intercellular signalling molecules (serotonin, dopamine, and acetylcholine). In contrast to glutamate, GABA, and melatonin, there is currently no convincing evidence suggesting that the second triad participates in intercellular signalling in plants. Finally, based on the details provided, we deduce putative principles concerning the recruitment of molecules for intercellular signalling.

## Signalling modes and signalling molecules

Here, we concentrate only on intercellular signalling rather than intracellular communication between different organelles. In animals, four different intercellular signalling modes exist: (i) paracrine; (ii) autocrine; (iii) endocrine; and (iv) gap-junction (which is animal-specific) (Alberts et al., 2019). In the paracrine mode, a signalling molecule produced by one cell is secreted into the intercellular space. The message is received by a second nearby cell and induces downstream physiological events through a signalling cascade mechanism. The autocrine mode is a subset of the paracrine where the sender is also the receiver. In the endocrine mode, the signalling molecules are transported over large distances through specialised vascular tissue to induce a reaction at far-distance parts of the multicellular organism. In gap-junction signalling, the signalling molecules shuttle between the sender and receiver cells through protein channels that directly connect the cytoplasm of these two cells.

**Figure 1.**
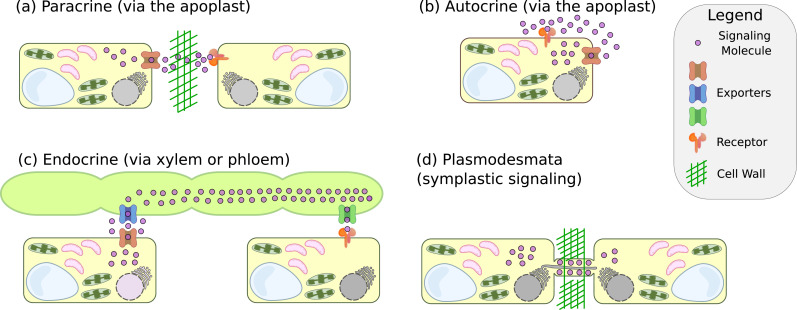
Modes of intercellular signalling. (**a**) Paracrine signalling, where cell-to-cell communication is mediated through the apoplastic space. (**b**) Autocrine signalling - similar to paracrine signalling, but when the sender cell also acts as a receiver cell. (**c**) Long-distance endocrine signalling that is mediated by translocation of the signalling molecules through the vascular tissue of plants. (**d**) Direct cell-to-cell communication and signalling through the plasmodesmata.

Plants utilise paracrine-like, autocrine-like, and endocrine-like (in plants, signalling through the vasculature tissues) modes (see Figure 1a–c) but not gap-junction signalling due to the rigid cell wall. Instead, they use cell-to-cell communication through plasmodesmata – a plasma membrane bridge between cells where space is restricted by an endoplasmic reticulum protrusion (see Figure 1d; Lucas et al., 2009).

Given these communication modes, what qualifies a molecule as an intercellular signalling one? In the context of microbiology, a set of four criteria was suggested (Winzer, 2002): (i) the signalling molecule is produced under certain physiological conditions, changes in the environment, or at a specific developmental stage; (ii) the signalling molecule is secreted to the extracellular space and is recognised by a specific receptor; (iii) a specific response is generated in the receiving cell once a specific threshold of the intercellular signalling molecules is reached; and (iv) the response involves cellular processes that deviate from those that are involved in synthesising or degradation of the metabolite itself.

In light of the existence of the plasmodesmata nanopores in plants, one is instructed to drop the first part of criterion (ii) as a necessary condition for intercellular signalling. Naturally, for cell types that lack plasmodesmata, such as pollen or guard cells, the first part of criterion (ii) should still hold. The traditional view among microbe, animal, and plant scientists was that intercellular signalling involves specialised receptors (Downward, 2001; McCarty and Chory, 2000; Mulligan et al., 1997). The receptor can be an intra-membranal protein (in most cases) or an intracellular one for amphipathic signalling molecules. Even today, the importance of receptors for signalling in plants is usually stressed (Cheung et al., 2020). Nevertheless, advances in understanding biological intercellular signalling suggest that a receptor is not necessarily needed for initiating a signalling cascade (see examples in sulfhydration [Corpas et al., 2019; Paul and Snyder, 2018], nitrosothiol formation by nitric oxide [Sanz et al., 2015], and reactive oxygen species (ROS) [Waszczak et al., 2018]). Thus, a molecule can be considered an intercellular signalling molecule even if no dedicated receptor exists. Hence, for the sake of this article, we suggest that to be classified as an intercellular signalling molecule, it should be (i) produced by a sender cell as a result of a specific environmental cue, under certain physiological conditions or a developmental stage above its homeostatic concentration, (ii) induce in a receiver cell or one of its organelles a response that deviates from a mere production or degradation of that specific molecule. The receiver cell, of course, can also be the sender cell (for the autocrine mode). However, in that case, the signalling molecule should, at least, be secreted from the symplast of the producing cell to the intercellular space (apoplast) and either interact with a receptor or be imported back to the symplastic space.

In animals, both GABA and glutamate act in a paracrine signalling mode, while melatonin acts more via an endocrine mode. All three molecules act via dedicated receptors. To assess the signalling function of this triad in plants and consider in what ways their intercellular signalling mode is similar or different from that in animals, (i) we will survey evidence for their participation in induced physiological response mechanisms; (ii) ask whether they act through paracrine or any other signalling mode; (iii) evaluate evidence for the existence of dedicated receptors; and (iv) finally, speculate what was the evolutionary driving force for their recruitment for intercellular signalling.

## GABA and glutamate

γ-Aminobutyric acid (GABA) and glutamate are four-carbon non-proteinogenic and five-carbon proteinogenic amino acids related to each other by one enzymatic step. Both are important plant metabolites. Both of them play an essential role in balancing metabolic pathways. For example, glutamate functions as a key component in the nitrogen fixation pathway and amino acid synthesis, and GABA is utilised for fixed nitrogen storage and balancing the carbon to nitrogen ratio (C/N ratio). Here we review the evidence for their intercellular signalling role in plants and hypothesise why they were adapted for this function.

## Glutamate

**Figure 2.**
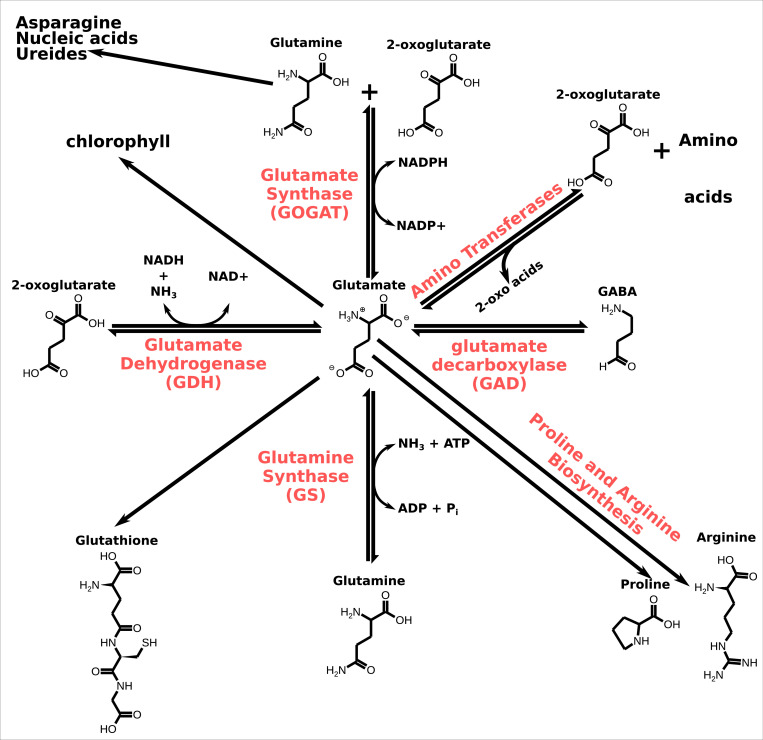
Glutamate metabolism. In red, the names of the enzymes that catalyse the chemical conversion. Similar reactions in the glutamate metabolic network also occur in animals (see Schousboe et al., 2014). Note that the figure does not show the balance of the chemical reactions.

Glutamate is located at the centre of an intricate metabolic network (see Figure 2; Forde and Lea, 2007). As consequences of its metabolic function, plants tend to control their glutamate concentration tightly (Forde and Lea, 2007). Thus, though endogenous glutamate concentration can slightly change under different physiological conditions, the glutamate concentration usually varies less than other amino acids in the glutamate metabolic network. Glutamate has been implicated in biotic (Kadotani et al., 2016; Kwaaitaal et al., 2011) and abiotic (Jin et al., 2019; Li et al., 2019; Yang et al., 2020) defence mechanisms as well as in fruit ripening (Snowden et al., 2015). In some cases, the glutamate protective effect is related to a broader protective effect of multiple amino acids (Cuin and Shabala, 2007; Goto et al., 2020), or their function in reporting nitrogen status (Liu et al., 2021). Transcriptome analysis in rice showed that exogenous glutamate treatment could result in a unique pattern of differential gene regulation relative to other organic (glutamine) or inorganic nitrogen sources (Kan et al., 2017). In other studies, exogenous glutamate-regulated genes were mostly related to metabolism or stress–response mechanisms (Gutiérrez et al., 2008; Ma et al., 2020). In addition to these functions, glutamate has possible intercellular signalling functions associated with its interaction with the glutamate receptor-like (GLRs) channels. This intercellular signalling function is associated particularly with the wounding response. Finally, glutamate acts in environmental-plant signalling. For general reviews about glutamate, see Liao et al., 2022; Qiu et al., 2019.

### Glutamate receptor-like proteins

**Figure 3.**
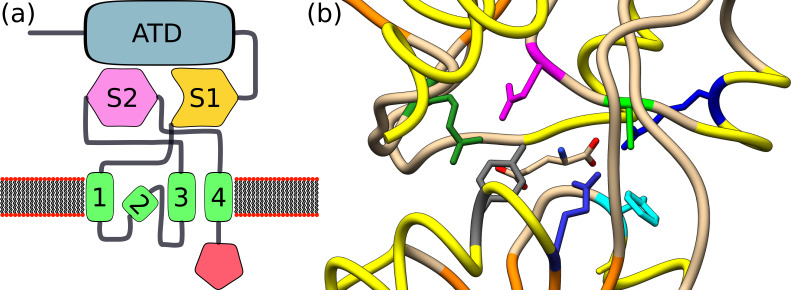
Glutamate receptor-like (GLRs), their agonists, and animal ionotropic glutamate receptors. (**a**) General structure of the ionotropic glutamate receptor. An ionotropic glutamate receptor (iGluR) monomer is composed of an amino-terminal domain (ATD) followed by the first half of the ligand-binding domain (S1). S1 is connected to three transmembrane segments (shown in green and numbered 1–3). The second transmembrane segment spans the membrane only partly. The Third transmembrane segment is connected to the second half of the ligand-binding domain. Finally, a fourth transmembrane segment is connected to the intracellular C-terminal domain. The figure is based on De Bortoli et al., 2016. Plant GLRs show a similar structure with a highly diverse C-terminal domain, three membrane-spanning segments (plus one partly spanning the membrane), and the divided ligand-binding domain. However, clade III ATD shows more homology to members of the sub-family C of G-protein-coupled receptors (GPCRs), which contain metabotropic glutamate receptors (mGluRs) and γ-aminobutyric acid B (GABA_B_) receptors in animals (Wudick et al., 2018a). (**b**) Crystal structure of the binding pocket of *Arabidopsis* GLR3.3 with glutamate as a ligand-based on Alfieri et al., 2020 (Protein Data Bank code: 6R85). Glutamate is depicted in sticks. Residues that were implicated in binding glutamate are Phe133 in cyan, Arg88 inbBlue, Ala83 in green, Arg11 in forest green, Tyr180 in grey, Glu177 in medium blue, and Asp81 in magenta. Backbone – in yellow α-helices, in orange β-strands.

Animal cells, particularly neurons, possess a family of non-selective cation channels gated by glutamate and glycine called ionotropic glutamate receptors (iGluRs) (Weiland et al., 2016). Plants possess a homologue family of membrane channel proteins called glutamate receptor-like (GLRs) (see Figure 3a; De Bortoli et al., 2016; Lam et al., 1998). *Arabidopsis* and tomato have 20 and 13 GLRs, respectively, divided into three clades (though GLRs from tomato clade I are not paralogues of *Arabidopsis’* clade I GLRs) (Aouini et al., 2012; De Bortoli et al., 2016). In *Oryza sativa* (rice), 16 GLR sequences were identified and were divided into four clades (the fourth clade also exist in other monocots) (De Bortoli et al., 2016). A more recent analysis based on bioinformatic tools suggested the existence of 26 genes in rice that are related to the *Arabidopsis* GLRs. However, 3D and transmembrane domain predictions proposed that only 10–12 out of these actually show a GLR-like domain organisation (Shi et al., 2022). Nevertheless, also in this case, the rice GLRs were divided into four subgroups. Despite the homology between animal iGluRs and plant GLRs, considerable differences exist between the two protein families (Wudick et al., 2018a). In particular, the ligand-binding domains, the pore domains, and the gate peptides show many differences between animal iGluRs and plant GLRs (Green et al., 2021). Nevertheless, general antagonists of iGluRs can influence plant growth (Singh and Chang, 2018) or the cytosolic concentration of Ca^2+^ (Dubos et al., 2003), which suggests interactions with GLRs. In fact, a homologous receptor called iGluR0 already exists in prokaryotes (Wudick et al., 2018a). All iGluRs and GLRs probably diversify from this origin.

In vascular plants, GLRs are suggested to participate in multiple physiological processes, including carbon and nitrogen sensing (Kang and Turano, 2003), root gravitropism (Miller et al., 2010), abscisic acid (ABA) signalling during germination (Kong et al., 2015), lateral root initiation and root development in rice (Li et al., 2005), abiotic and biotic response (Cheng et al., 2018; Li et al., 2013; Wang et al., 2019b), stomatal closure (Cho et al., 2009; Kong et al., 2016), and pollen tube growth (Michard et al., 2011). Moreover, in *Arabidopsis*, GLRs are expressed in different organs and tissues, though different GLRs have different expression patterns (Chiu et al., 2002; Roy et al., 2008). However, these GLRs functions are not necessarily related to glutamate.

Why? When comparing iGluRs to GLRs, one must analyse both the GLR ion selectivity and the agonist repertoire. Since Ca^2+^ signalling is essential for plant physiology (Choi et al., 2016; Demidchik et al., 2018; Tian et al., 2020), calcium influx can connect GLRs signalling to physiological events. In vascular plants, the cytoplasmic-free Ca^2+^ concentration is kept at a very low value (50–150 nM), while the concentration in the apoplast and internal organelles can reach 0.1–10 mM (Demidchik et al., 2018). Thus, the opening of a Ca^2+^-permeable channel can be a vital message transducer to physiological functions in the cytoplasm (and the nucleus). Indeed, most GLRs (e.g. AtGLR3.3 and AtGLR3.6; Shao et al., 2020) have a high permeability to Ca^2+^. Nevertheless, in *Arabidopsis*, only for some GLRs, such as AtGLR1.4, AtGLR3.4, and AtGLR3.4/AtGLR3.2, evidence from a heterologous expression system suggests a predominantly Ca^2+^ permeability (Tapken et al., 2013; Vincill et al., 2012; Vincill et al., 2013).

Concerning their agonists, GLRs, unlike iGluRs, have a broad ligand repertoire (Gangwar et al., 2021). For example, for AtGLR1.4 (Tapken et al., 2013), AtGLR1.2 (Michard et al., 2011), AtGLR3.2 (Gangwar et al., 2021), OsGLR3.4 (Yu et al., 2022), and some other cases (Kong et al., 2016), evidence suggests that glutamate is not the primary physiological agonist. By contrast, for AtGLR3.3, AtGLR3.6, and AtGLR3.4, there is evidence that glutamate is an important agonist (see below). Thus, though in some cases plants show a unique response to glutamate over other amino acids, in other cases, glutamate was not the most potent agonist to specific GLRs under study (Alfieri et al., 2020; Dubos et al., 2003; Mou et al., 2020; Vincill et al., 2013). It has been suggested that GLRs might have evolved to act as general nitrogen state sensors (Gent and Forde, 2017; Kang and Turano, 2003). In other words, though both protein families possess a common bacterial ancestor (De Bortoli et al., 2016), they embarked on somewhat different evolutionary courses.

### Glutamate intercellular signalling

Several studies have shown that glutamate (in a concentration of several hundreds of μM) can elicit (transient or continuous) cation current through the plasma membrane in plant cells (Dennison and Spalding, 2000; Meyerhoff et al., 2005; Qi et al., 2006), transient outward current composed of sodium efflux and chloride influx (Demidchik et al., 2004), or even propagating action potential (Felle and Zimmermann, 2007; Stolarz et al., 2010). Similarly, glutamate can induce biotic-stress-related nitric oxide (NO) production (Vatsa et al., 2011). In these cases, a GLR-dependent calcium influx was usually implicated but not definitely proven.

As of today, only AtGLR3.3, AtGLR3.6, and AtGLR3.4 have been directly shown to be gated by glutamate (Green et al., 2021; Qi et al., 2006; Shao et al., 2020). However, even for AtGLR3.3 (Li et al., 2013; Alfieri et al., 2020) and AtGLR3.4 (Vincill et al., 2013; Grenzi et al., 2021), evidence suggests that other amino acids have a lower dissociation constant than glutamate or that cooperative binding of glutamate with other metabolites results in a much more extensive response (see Figure 3b). Since AtGLR3.5 has a very similar ligand-binding site to AtGLR3.3, it might also be gated by glutamate to some extent. In fact, it was shown that glutamate could induce stomatal closure in *Arabidopsis* and fava beans (*Vicia faba*) in an AtGLR3.5-dependent manner (Yoshida et al., 2016). However, AtGLR3.5 was reported to be mainly a methionine-activated cation channel (Kong et al., 2016). Furthermore, AtGLR3.7 might also be somewhat gated by glutamate (Yoshida et al., 2016). Finally, there is evidence that glutamate can rescue an *antiAtGLR1.1* line (Kang and Turano, 2003).

**Figure 4.**
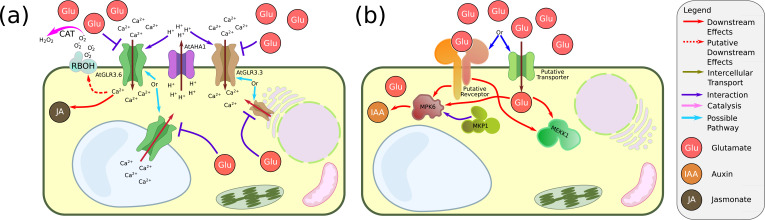
Glutamate signalling in plants. (**a**) Glutamate signalling via glutamate receptor-likes (GLRs). It was suggested that, following wounding, glutamate is secreted from the phloem. In parallel, the ATPase AtAHA1 is deactivated, and the apoplast pH rises. These processes activate AtGLR3.3 or AtGLR3.6 (or both) and cause electric potential propagation (not shown) as well as Ca^2+^ influx to the cytoplasm. The activation of AtGLR3.3 and AtGLR3.6 initiates a signalling cascade that results in jasmonate synthesis. In some plants (e.g. tomato), it might also lead to H_2_O_2_ signalling via RESPIRATORY BURST OXIDASE HOMOLOG (RBOH). Note that AtGLR3.3 and AtGLR3.6 are not localised to the same cell populations in the leaves (phloem sieve elements and xylem contact cells, respectively). It was also found that they might not localise to the plasma membrane (ER and vacuole, respectively). (**b**) Glutamate in environmental-root tip signalling. Putative glutamate signalling during RSA restructuring. A putative receptor or a putative transporter (not identified yet) transduces the presence of extracellular glutamate to the cytoplasm. Downstream, both MEKK1 and the pair MKP1/MPK6 are influenced. Even downstream from MKP1/MPK6, the level of auxin is modified.

The most substantial evidence for glutamate acting as an intercellular signalling molecule in a GLR-dependent manner is related to AtGLR3.3 and AtGLR3.6 participation in the wounding response. When *Arabidopsis* leaves are wounded (e.g. by herbivorous insects), both Ca^2+^ waves and systemic electric potential signals propagate from the wounded site to other parts of the leaf and systemically connected leaves to induce the synthesis of jasmonate, the phytohormone that controls the defence response (Mousavi et al., 2013). The propagation of these signals depends on AtGLR3.3 and AtGLR3.6, and in a *glr3.3/glr3.6* mutant, the signals did not propagate beyond the wounded leaf. Similarly, in tomato, *SlGLR3.1* and *SlGLR3.5* (the tomato homologue of *AtGLR3.6*) were implicated in the jasmonate synthesis following root-nematode attack (Wang et al., 2019a). Further, it was shown that AtGLR3.3 localises to the phloem sieve elements, while AtGLR3.6 localises to the xylem contact cells, and both cell-type populations are needed for the propagation of the systemic electric potential signal, which precedes the Ca^2+^ wave (Nguyen et al., 2018; Toyota et al., 2018). Consequently, AtGLR3.3 and AtGLR3.6 show different functions concerning the actual amplitude and duration of the wound-induced electric signal (Salvador-Recatalà, 2016). Glutamate accumulates in the wounded region and propagates to other regions of the same leaf (Toyota et al., 2018). It was also shown that exogenous glutamate at a very high concentration (100 mM) could induce an *AtGLR3.6* (and *AtGLR3.3*)-dependent long-distance Ca^2+^ signalling between different leaves (Toyota et al., 2018). Thus, the model suggests that, upon wounding, glutamate is secreted from the phloem, where it is usually found at a high concentration (10–50 mM) (Hunt et al., 2010), to the apoplast, where its resting concentration is about 1 mM. Since the dissociation constant of glutamate to GLR3.3 is in the low micromolar range, this elevation of concentration activates GLR3.3 and GLR3.6 and induces the Ca^2+^ and electric responses (Alfieri et al., 2020; Grenzi et al., 2021). Additionally, during root-to-leaf wounding signalling, H^+^ was also needed to deactivate the GLRs after the Ca^2+^ wave passed the cells (Shao et al., 2020). Hence, a current, somewhat more intricate model, suggests that a still unrecognised signal inhibits H^+^-ATPase 1 (AHA1). Following AHA1 deactivation, the apoplastic space becomes more alkaline. Then, together with gating by phloem-secreted glutamate, Ca^2+^ flows via GLR3.3 and GLR3.6, which causes a downstream signalling cascade. Subsequently, AHA1 is reactivated, the apoplast acidifies, and the GLRs are shut off (see Figure 4a).

Note that detection of AtGLR3.3 and AtGLR3.6 coupled to a fluorescent protein suggests that they do not mainly localise to the plasma membrane but to the vacuole and endoplasmic reticulum (Nguyen et al., 2018). Moreover, it was found that auxiliary proteins (CORNICHON) are responsible for sorting iGluRs and GLRs (including AtGLR3.3) in vivo (Wudick et al., 2018b). Thus, the localisation of fluorescence-tagged GLRs to the plasma membrane observed in some studies might not represent their actual localisation. Hence, an additional step of glutamate import to the cytoplasm might be needed before the activating of AtGLR3.3 or AtGLR3.6 can occur. Moreover, also AtGLR3.1 and AtGLR3.5 were implicated in the wound-response signalling (Mousavi et al., 2013; Salvador-Recatalà, 2016). In particular, AtGLR3.5 may act as an on/off switch to prevent the propagation of the electrical signals to non-systemically connected leaves. However, it is unclear whether AtGLR3.5 and AtGLR3.1 functions are related to the glutamate one.

It is still unclear what is the relevant concentration of glutamate needed for the activation of GLR3.3 and GLR3.6 (Grenzi et al., 2021). On the one hand, the equilibrium dissociation constant (K_d_) of several GLRs to glutamate was in the micromolar range. On the other hand, a glutamate concentration of tens of millimolar was needed to induce long-distance glutamate-dependent signalling. Several hypotheses attempt to suggest an explanation for this discrepancy. These hypotheses include the involvement of protons in GLR3.3 and GLR3.6 activation, the involvement of CORNICHON protein in the channel opening, or even the implication of changes in the xylem hydrostatic pressure (Moe-Lange et al., 2021; Shao et al., 2020; Wudick et al., 2018b). Another possible explanation may involve the localisation of the GLRs. If GLR3.3 and GLR3.6 are intracellular proteins and do not localise to the plasma membrane (Nguyen et al., 2018), a relatively high extracellular concentration will probably be needed for their activation.

In addition to the participation of glutamate in the wounding response, there was one report that associated it with another stress response that might suggest a signalling function. In the past, glutamate was linked to plant response to aluminium (Al^3+^) stress-induced root growth inhibition (Sivaguru et al., 2003). In that case, exogenous glutamate (5 mM) elicited a fast (≈2 min) depolymerisation of cortical microtubules. The authors suggested a similar mechanism where Al^3+^ stress causes glutamate exudation, followed by GLRs activation and downstream physiological effects. Which GLR (if any) is responsible for this effect remains unknown.

### Glutamate and the root system architecture

Glutamate (or glutamic acid) is an abundant molecule in some soils (Li et al., 2014c; Rothstein, 2009). Hence, it was suggested that glutamate could act in soil-root signalling at the root tip (Forde, 2014; Forde and Walch-Liu, 2009), see also Figure 4b. In *Arabidopsis*, exogenous glutamate can inhibit primary root growth in low nitrate media via the root tip even in the presence of glutamine (Walch-Liu et al., 2006). Nitrate, acting as a signal at the root tip, abolishes the glutamate primary root inhibition effect (Walch-Liu and Forde, 2008). At least two mitogen-activated protein kinase (MAPK) pathways were implicated in this process (Forde et al., 2013; López-Bucio et al., 2018). It was also suggested that auxin is acting downstream of glutamate (Walch-Liu et al., 2006), and it might be the case that auxin acts downstream from one of these MAPK pathways. Nevertheless, whether this glutamate effect is mediated through GLRs is still an open question. There is currently some genetic evidence suggesting the involvement of *Atglr2.5* and *Atglr2.6* in this process (Walch-Liu et al., 2017). However, several biochemical assays failed to find supporting evidence for GLRs' involvement in the process (Forde et al., 2013; Walch-Liu et al., 2006). Interestingly, AtGLR3.6 and its rice homologue OsGLR3.1 are involved in structuring the root system architecture (RSA) probably by acting upstream of the auxin level (Li et al., 2005; Singh et al., 2016). However, it is unclear whether these effects are related to glutamate itself.

Note that, in this case, the signalling is not an intercellular signalling process per se, according to the criterion developed above, but signalling from the environment into the plant. Nevertheless, it is still interesting to try to understand the mechanistic relationships between glutamate acting in RSA and that in intercellular signalling. Similarly, it will be interesting to consider the evolutionary origin of the dual functionality of glutamate as a signal. One possibility is that the original role of glutamate was in environment-to-plant signalling, and glutamate was subsequently co-opted into intercellular signalling. We dwell a bit more on this hypothesis in the ‘Discussion’. Naturally, the opposite option is also possible, or perhaps these two processes evolved simultaneously. In any case, more evidence is needed to identify which of these options is the correct one.

### Glutamate intercellular signalling: Summary

To summarise, glutamate acts as a GLRs-dependent intercellular signalling molecule linked to Ca^2+^ signalling during root and shoot wounding. In these cases, glutamate acts through a paracrine signalling mode. Glutamate might also act as a GLR-related intercellular signalling molecule during other physiological conditions, but these conditions are left to be found.

Why did glutamate become an intercellular signalling molecule in plants (and probably also in animals)? Glutamate is a primary metabolite with crucial functions in the metabolic network and particularly in nitrogen assimilation. Moreover, glutamate is one of the most abundant metabolites. Hence, cells developed intricate mechanisms for sensing the glutamate concentration (for a review, see Moroz et al., 2021). While glutamate cannot diffuse through the cell membrane, a receptor for glutamate (iGluR0) exists already in bacteria, so glutamate functions as an intercellular signalling already in bacteria. Thus, when plants started to develop, they possessed an already existing machinery that could be utilised for intercellular signalling.

Moreover, the pivotal role of glutamate in soils was also being associated with the development of other mechanisms to shuttle glutamate across the plasma membrane. Indeed, in angiosperms, glutamate can also be transported over the plasma membrane by dedicated transporters. In *Arabidopsis,* several transporter families were identified for amino acid transport (Dinkeloo et al., 2018; Tegeder and Masclaux-Daubresse, 2018). The importing rate of amino acids depends on the tissue type, concentration of the amino acid, and developmental stage (for a review see, Yao et al., 2020). Particularly, evidence exists for the involvement of the amino acid permease AAP1 in the uptake of glutamate from the soil at naturally occurring concentrations (Perchlik et al., 2014). In addition, a complex mechanism is implicated in the transport of amino acids, in general, and glutamate, in particular, via the xylem and phloem. Several transporters from the UmamiT family were shown to act as efflux transporters (though the transport directionality depends on the electrochemical gradient) (Müller et al., 2015). Though these transporters are not involved in glutamate signalling per se, their presence might have acted as an additional evolutionary force in the recruitment of glutamate for intercellular signalling in plants.

Thus, since (i) glutamate has a pivotal function in the metabolic network and its concentration is tightly regulated and sensed, and since (ii) the existence of auxiliary machinery to transfer information across the plasma membrane was in place, glutamate became a good candidate to act as an intercellular signalling molecule in the context of multicellular life (for both plant and animal cells). An instructive parallel example from the plant world showing how glutamate’s pivotal place in the metabolic network probably instructed its recruitment for environment-to-root signalling was also discussed. This ability to use GLRs (or iGLuR) for signalling substantially diversified in plants and animals compared to their prokaryotic origin. In mammals, iGluRs function probably was restricted to signalling in the nervous system, while in plants, GLRs were recruited for multiple glutamate-independent processes.

## GABA

**Figure 5.**
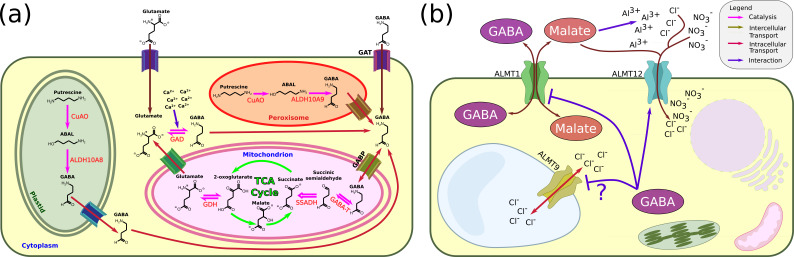
GABA in plant physiology and signalling. (**a**) GABA synthesis (based on Fait et al., 2008 and Gilliham and Tyerman, 2016). GABA is synthesised via two main routes. In the context of the GABA shunt, it is synthesised in the cytoplasm from glutamate by glutamate decarboxylase (GAD) and is then imported to the mitochondria, where it is converted to succinic semialdehyde. In the plastids and the peroxisomes, it is synthesised from the diamine putrescine. The ability of the GABA shunt to control the C/N ratio makes GABA a pivotal metabolite under various stress conditions. Similarly, the importance of polyamines in stress mechanisms also connects GABA to the maintenance of cell physiology. In addition, since the GAD reaction consumes a proton, GABA might act as a pH regulator. (**b**) GABA in signalling. Suggested GABA signalling events depend on the ALMT protein family. In the roots, GABA inhibits the transport of malate through ALMT1 upon Al^3+^ stress. In the leaves, GABA might regulate stomatal closure during drought by inhibiting Cl− transport through the tonoplast localised ALMT9 (which does not count as an intercellular signalling event by our criteria). GABA can also regulate stomatal closure through ALMT12. However, the precise physiological relevance of this process is still not clear. GABA inhibition of ALMT1 malate transport might be achieved through an interaction with a peptide moiety at the cytoplasmic side. It is not known whether this is achieved primarily by GABA imported to the cell via ALMT1 itself or by endogenous cytoplasmic GABA. The possible involvement of GABA in pollen tube growth is not shown since the underlying mechanism is not yet deciphered. Similarly, the putative interactions between GABA and GORK is not shown. GAD, glutamate decarboxylase; SSR, semialdehyde reductase; GABA-T, GABA transaminase; SSADH, succinic semialdehyde dehydrogenase; GABP, mitochondrial GABA transporter; GAT, GABA transporters; CuAO, copper-containing amine oxidase; ALDH, aldehyde dehydrogenase.

GABA is an important metabolite that contributes to plant cell physiology (Shelp et al., 2017; Ramos-Ruiz et al., 2019; Kaspal et al., 2021; Xu et al., 2021b). One of its primary functions is related to the GABA shunt that contributes to the cell carbon/nitrogen ratio (Michaeli and Fromm, 2015). The GABA shunt, a sequence of enzymatic reactions, starts with the channelling of 2-oxoglutarate carbon from the tricarboxylic acid (TCA) cycle to produce glutamate. Subsequently, GABA is synthesised from glutamate by glutamate decarboxylase (GAD). Most plant *gad* genes possess a calmodulin (CaM) moiety activated by Ca^2+^ (Michaeli and Fromm, 2015). The GABA shunt is closed through GABA conversion to succinic semialdehyde (SSA) by GABA-T (also known as POP2) and further to succinic acid by succinic semialdehyde dehydrogenase (SSADH) (see Figure 5a). A second route for GABA synthesis is through polyamine catabolism (Shelp and Zarei, 2017). In that case, the diamine putrescine is oxidised by amine oxidase (CuAO) to 4-aminobutanol (ABAL). Subsequently, aminoaldehyde dehydrogenase converts ABAL to GABA (Zarei et al., 2016).

As both polyamines and Ca^2+^ signalling are involved in various stress–response mechanisms, it is predictable that GABA acts under various stress conditions (Kinnersley and Turano, 2000). Indeed, GABA (and especially exogenous GABA) was shown to influence adaptation to heat (Li et al., 2016a), cold (Malekzadeh et al., 2013), salt (Wang et al., 2017a), drought (Li et al., 2016b), hypoxia (Mustroph et al., 2014; Wang et al., 2014), heavy metals (Seifikalhor et al., 2020), UV (AlQuraan, 2015), and iron deficiency (Guo et al., 2020). Similarly, exogenous GABA can alleviate the effects of various deleterious abiotic conditions (Li et al., 2020b). In some cases, evidence exists for a differential level or activity of GABA-synthesis enzymes that result in the accumulation of endogenous GABA (Kaplan et al., 2007). In these cases, GABA concentration can increase by up to 2700% (Bown and Shelp, 2020). Exogenous GABA can also change the endogenous GABA concentration since GABA can be imported to the cell through GAT1 and other amino acid transporters (Meyer et al., 2006). In addition, GABA accumulation during stress can protect plants through its influence on pH buffering (Shelp et al., 1999). The proposed mechanism involves consuming H^+^ during GABA production by GAD. Thus, the accumulation of GABA during stress–response mechanisms can help buffer cytosolic acidification.

In some cases, the protective effect of GABA was correlated with the mitigation of ROS accumulation and H_2_O_2_ signalling modulation (Shabala et al., 2014; Shi et al., 2010). In other cases, endogenous (Renault et al., 2012, Renault et al., 2011) or exogenous (Ji et al., 2018; Lancien and Roberts, 2006) GABA is also related to changes at the transcriptional and metabolic levels.

Some studies reported that exogenous GABA affects the RSA even when applied at a concentration that does not cause a significant GABA accumulation. For example, exogenous GABA could inhibit *Arabidopsis* primary root growth (Barbosa et al., 2010). Similarly, exogenous GABA treatment to poplar leaf explants resulted in a time delay of adventitious root formation (Xie et al., 2020). Other functions that were implicated in GABA influence on plants included carbon–nitrogen interactions (Batushansky et al., 2015; Chen et al., 2020; Michaeli et al., 2011), leaf nitrate metabolism (Li et al., 2018), and senescence (Jalil et al., 2017). Next, we review evidence for the participation of GABA in intercellular signalling in plants and suggest a possible reason why it was recruited for such a role.

### GABA and signalling

Over the years, multiple suggestions have been made that GABA has a double role in plants (i) as a critical metabolite and (ii) as an intercellular signalling molecule (Bouché and Fromm, 2004; Bown and Shelp, 2016; Gilliham and Tyerman, 2016). Nevertheless, for many years the function of GABA as an intercellular signalling molecule in plants was seriously questioned (Batushansky et al., 2014). As of today, three processes might suggest an intercellular signalling function of GABA in plants. In addition, some evidence also connects GABA to signalling during hypoxia. Here, we discuss each one of them separately. For a recent comprehensive review about GABA signalling in plants, see Fromm, 2020; Xu et al., 2021b.

### GABA signalling and the aluminium-activated malate transporter

Several years ago, a putative GABA receptor – ALMT1 – was identified in plants (Ramesh et al., 2015). This receptor belongs to the anion channel aluminium-activated malate transporter (ALMT) family (for a review of the ALMT family, see Sharma et al., 2016). Its molecular structure was recently resolved, providing a putative molecular mechanism for its malate selectivity and aluminium gating (Wang et al., 2021a). Some members of the ALMT channel family, such as ALMT1, are involved in the response to Al^3+^ stress, while others are not. Several members of the ALMT family can also interact with GABA (see below), but in other cases (e.g. OsALMT4) the ALMT channel is unlikely to be a GABA receptor (Liu et al., 2018). ALMTs are not homo8logues of the animal GABA receptor. The only homology between animal GABA_A_ ion-channel receptors and ALMT resides in a 12 amino acid motif (Ramesh et al., 2015). A homologue of this peptide is found in most ALMT proteins (Ramesh et al., 2016). It was hypothesised that the GABA interaction with members of the ALMT family occurs at this site.

In the roots, one of the main effects of GABA is the regulation of primary root growth in the presence of Al^3+^ (Ramesh et al., 2015). To protect against Al^3+^ stress, plants exude malate that chelates the Al^3+^ ions (Ramesh et al., 2015). GABA can regulate malate exudation. That is, there is an inverse linear relationship between endogenous GABA concentration and malate transport through ALMT1 (Ramesh et al., 2018). Similarly, GABA concentration is inversely correlated to the inhibition of primary root growth under Al^3+^ stress. Thus, GABA can inhibit malate transport through the ALMT1 channel (see Figure 5b). ALMT1 not only functions as a malate transporter and GABA receptor. In fact, GABA itself can also be imported from the apoplast or exported into the apoplast via the ALMT1 channel, depending on the GABA gradient direction (Ramesh et al., 2018). Functionally, GABA inhibits anion transport through ALMT1 channels from the cytoplasmic side by regulating the opening time of the channel (Long et al., 2019). The interaction between GABA and ALMT1 channels, resulting in ion transport inhibition and membrane potential hyperpolarisation, can serve as a general mechanism for GABA intercellular signalling in plants in an autocrine-like (or maybe paracrine-like) signalling mode.

However, there is a crucial difference in the way that GABA modifies the membrane potential in nervous and plant roots cells. In the nervous cells of adult mammals, the primary ions responsible for generating the membrane potential are Na^+^ and K^+^ (Lazzari-Dean et al., 2021). In that case, the membrane depolarisation depends on Na^+^ influx, while repolarisation and hyperpolarisation are created by K^+^ efflux. Concomitantly, an increased concentration of post-synaptic GABA induces the opening of the GABA_A_-chloride channels and increases Cl^-^ influx. Consequently, membrane hyperpolarisation is increased, and excitability is reduced. By contrast, in plant roots, Cl^-^ concentration is higher in the cytoplasm than in the apoplast (Geilfus, 2018). Hence, like in the nervous cells of adult mammals, GABA interaction with ALMT1 also reduces membrane depolarisation. However, in this case, the effect is achieved by inhibiting the Cl^-^ efflux through ALMT1 (Ramesh et al., 2015).

### GABA and stomata regulation

A function for GABA was implicated in stomatal-aperture regulations (see Figure 5b; Mekonnen et al., 2016). Mutant *Arabidopsis gad1/2* plants that have a reduced concentration of GABA were oversensitive to drought. This drought sensitivity was a consequence of a larger stomatal aperture, which was accompanied by a reduction in the endogenous GABA concentration. It is known that two ALMT are expressed in *Arabidopsis* leaves. One of them – ALMT12 – is a plasma membrane channel, while the other – ALMT9 – is a tonoplast channel (Sharma et al., 2016). Recently, a GABA-dependent function for both channels was suggested in stomatal-aperture regulations (Xu et al., 2021a). In that case, both exogenous GABA and muscimol (a GABA homologue) were able to suppress light-induced stomatal opening and dark-induced stomatal closing in *Arabidopsis* epidermal peels. Both of these metabolites were also able to suppress ABA and H_2_O_2_-induced stomatal closure. When comparing a *gad2* mutant to an *almt9/gad2* mutant, it was found that while the *gad2* mutant had larger stomatal pores (and hence a larger rate of gas exchange and transpiration), the *almt9/gad2* mutant showed a wild-type-like phenotype. Moreover, *almt9* mutant expressing ALMT9F234C/Y245C (a mutant with a double mutation in the presumed GABA binding site) behaved like the *gad2* mutant. Based on this evidence, the authors of that work suggested that GABA acts as a ‘brake’ or a fine modulator of stomatal opening and closing due to its regulation of the ion flow to and from the vacuole across the tonoplast through ALMT9. In this case, the signalling cascade is fully intracellular since GABA is produced in the guard cells and regulate their own behaviour without leaving the cell. Indeed, expressing a GAD in a related cell population (spongy mesophyll cells) under a constitutionally active promoter increased the concentration of GABA in the leaves but did not show the GABA-related phenotype (Xu et al., 2021a). In contrast, expression of *GAD2* in guard cells under the guard cell-specific promoter (GC1::GAD2) was able to restore stomatal conductance to the wild level, but only under drought conditions and not under standard conditions. Thus, it needs to be clarified whether GABA-related stomatal responses depend solely on the concentration of GABA in the guard cells. Currently, this work mainly provides evidence for intracellular but not intercellular GABA-related signalling in plants. Since intercellular signalling is centred on communication (even if it is self-communication), the function of an intercellular signalling molecule necessitates the passage of the signal (but not necessarily the signalling molecule itself) over the plasma membrane, which is not the case for the ALMT9-dependent signalling in plants.

Note that, a second study failed to detect GABA-mediated anion transport through ALMT9 in a patch-clamp experiment when GABA was applied from the cytosolic or the vacuole sides (Jaślan and De Angeli, 2022). This study was, however, criticised by the authors of the GABA-stomatal regulation study based on the nature of the ALMT9 expressed (GFP tag), the cell type it was expressed in (mesophyll cells), and the nature of the orthologous system (*Nicotiana benthamiana*) (Gilliham and Xu, 2022). Hence, whether GABA acts directly or indirectly on ALMT9 is still an open question. Several options might explain these contradictory results. For example, one possibility is that GABA activates ALMT9 only in the presence of an unknown regulatory factor. Another option is that GABA influence on ALMT9 anion transport is mediated through its influence on metabolism rather than through direct binding.

However, in the study by Xu et al., 2021a, it was found that also *almt12* mutant, which is a plasma membrane channel mutant, was insensitive to exogenous GABA when transitioning from light to dark. Similarly, an *almt9 x almt12* was completely insensitive to GABA under light-induced opening and dark-induced stomatal closure. These facts suggest a possible additional role of GABA in intercellular stomatal signalling that was not elaborated on by the study under drought conditions. In that case, the intercellular signalling might be autocrine or a paracrine one. However, further research is needed to identify such relevant physiological conditions. Structural studies such as the recent one by Qin et al., 2022, deciphering the structure of ALMT12 and its interaction with malate (but not with GABA in that case) most probably can help in assigning the specific interaction mechanism of GABA and proteins from the ALMT family.

### GABA in pollen tube growth

Some evidence connects the GABA concentration of the pistil to the growth of the pollen tube. First, in *Arabidopsis*, a gradient of GABA exists in the female reproductive tracts, from the stigma through the style and the septum to the micropyle (Palanivelu et al., 2003). By contrast, in a *gaba-t* mutant with a higher cytoplasmic GABA concentration, the GABA gradient in the female reproductive tract is diminished (though it is maintained between the septum and the micropyle). Also, exogenous GABA can stimulate pollen tube growth at low GABA concentration (10–100 μM) and inhibit its growth at a higher GABA concentration. However, in the *gaba-t* mutant, exogenous GABA fails to stimulate pollen tube growth. Similar results were also observed in tobacco (Yu et al., 2014). In that case, it was also shown that exogenous GABA could initiate a calcium flux into pollen tube protoplasts. This cation flux was probably not mediated by a GLR channel. Moreover, both GAD1, CaM, and GABA were localised to the growing pollen tube tip. Finally, a GAD inhibitor caused aberrant pollen tube growth and caused both the actin filaments and the vesicle trafficking at the tip to become disorganised and random. Similar results were also observed in *Picea wilsonii* (Ling et al., 2013).

Some additional evidence for GABA involvement in pollen tube growth was obtained from a coupled bioinformatic transcriptional analysis of pollen tubes and the stigma (Yue et al., 2014). In that case, it was shown that enzymes of the GABA shunt are activated in the stigma. However, as of today, there is no direct evidence of GABA secretion from the female reproductive tracts to guide the pollen tube as paracrine signalling. It is known that the female reproductive tract secret high-energy metabolites for pollen tube growth (Yue et al., 2014). Hence, GABA might only act as a nitrogen source for pollen growth, or GABA itself is not the signal.

Nevertheless, some recent evidence supports the idea that GABA acts as an intracellular signalling molecule during pollen tube growth (Domingos et al., 2019). In that case, electrophysiological measurements of pollen tube protoplasts suggested that AtALMT12 is an important contributor to the oscillatory anion efflux at pollen tube tips, and an *almt12* pollen tube mutant showed a reduced anion current. Moreover, the addition of GABA to the cytoplasmic side resulted in anion current inhibition in wild-type *Arabidopsis* pollen tube protoplasts. Finally, muscimol inhibited pollen tube growth in wild-type *Arabidopsis* pollen tubes but did not affect pollen tube growth in an *almt12* mutant line. Thus, if GABA acts as an intercellular signalling molecule during pollen tube growth, it probably affects the growth through ALMT12 (and its close heterologous ALMT channels). In that case, since GABA inhibits anion current through ALMT12 from the cytoplasmic side, its intercellular signalling function will have to be supported by dedicated GABA importers. Further research is needed to clarify the exact role of GABA in pollen tube growth and its putative function as an intercellular signalling molecule.

### GABA and GORK

GORK (Guard Cells Outward Rectifying K^+^ Channel) is the major K^+^-selective outward rectifying channel in plants (Adem et al., 2020). It is involved in various physiological conditions where regulating the K^+^-efflux is needed, including most biotic stress conditions. GORK is a voltage-activated channel but can probably also be activated by ATP, cAMP, and cGMP. In recent years, some evidence suggested that GABA may also act as a ligand for GORK. If this is indeed the case, it may represent an additional GABA-dependent signalling route in plants independent of the ALMT family. Indeed, in *Arabidopsis*, comparing a *pop2* mutant to the wild type or *gad1,2* mutants suggested that the GABA level influences K^+^/Na^+^ balance under NaCl stress (Su et al., 2019). Similarly, under hypoxia, the K^+^-efflux from the root elongation zone was lower in the *pop2* mutant than in the *gad1,2* mutant, while the efflux from the wild type was intermediate between the two (Wu et al., 2021). This evidence suggested some relationship between GABA intercellular concentration and K^+^-efflux regulation. In addition, sequence analysis suggested some low homology in GORK to the conserved F...W...E...L peptide sequence in ALMT1, suggested to be the docking site for GABA based on its homology to the GABA_A_ receptor (Adem et al., 2020). It was also shown that GABA could induce a low K^+^-efflux from the root epidermis of *Arabidopsis*. However, it is not clear, in that case, whether GABA induces the K^+^-efflux through direct interaction with the GORK channel, and if it occurred, whether it would involve transduction of the GABA concentration status across the membrane. Hence, further research is needed to conclude whether the influence of GABA on the K^+^-efflux through GORK represents an intercellular signalling mechanism.

### GABA signalling: Summary

Currently, three to four cases might represent intercellular GABA signalling events. In the first, Al^3+^ stress in the root, GABA might act in a paracrine or an autocrine manner. In the second, in stomatal aperture regulation, there is some indication that GABA intercellular signalling might occur through ALMT12 in a paracrine-like or an autocrine-like manner. In the third case, there is some evidence for a paracrine-like GABA signalling process in pollen tube growth. However, the results are not conclusive, and direct evidence for such a process is still lacking. In addition, some evidence suggests the involvement of GABA in K^+^-efflux regulation under stress. However, more evidence is needed to prove or disprove this process as a GABA-dependent intercellular signalling process. Finally, some authors suggest that GABA acts as a long-distance signalling molecule through the phloem (not discussed in this article) (Beuve et al., 2004). However, some crucial arguments and experimental evidence were presented against this hypothesis (e.g. see Shelp, 2012; Scholz et al., 2017).

If GABA does act as an intercellular signalling molecule in plants, why was it recruited for this function? Similar to glutamate, GABA is also a key metabolite in maintaining the carbon/nitrogen ratio, in multiple stress responses, and possibility in pH homeostasis regulation. Thus, based on its metabolic centrality and similar to glutamate, we suggest that it had a high potential to be recruited for intercellular signalling. Indeed, in the first three cases discussed in plants, the putative role of GABA intercellular signalling is probably related to its metabolic function. That is, its nitrogen source character in pollen tube growth and the fact that its concentration is under tight regulation for metabolic purposes in stomatal closure and Al^3+^ stress. Presumably, similar biological forces help to shape GABA signalling in multicellular animals. Again, similar to glutamate, GABA cannot pass the plasma membrane passively. However, unlike glutamate, for GABA, no common receptor machinery of prokaryotic origin was identified in plants and animals. Hence, the machinery associated with information transfer evolved separately in these two kingdoms. While this machinery is well characterised in animals, we are now only starting to discover it in plants.

## Melatonin

In animals, melatonin acts both within and outside the central nervous system (CNS) and is associated with regulating the circadian clock and anti-inflammatory free radical scavenging (Bubenik, 2008; Esposito and Cuzzocrea, 2010). In plants, melatonin biosynthesis pathway was primarily identified in rice as a multi-enzymatic process starting from tryptophan (Zhao et al., 2019; Tan and Reiter, 2020). Some synthesis steps leading to melatonin from tryptophan in plants are similar to those in animals, while others are unique (Zhao et al., 2019; Tan and Reiter, 2020). In plants, melatonin has profound effects on plant physiology. For extensive reviews, see Arnao and Hernández-Ruiz, 2017b; Wang et al., 2017b; Erland and Saxena, 2018; Back, 2021; Zhao et al., 2021. Moreover, the interest among plant scientists in melatonin has intensified in recent years, as is evident, for example, from the recent issue of the *Journal of Experimental Botany* (volume 73, issue 17, 2022) that was devoted to this metabolite. However, the assignment of melatonin as an intracellular signalling molecule is incomplete, and the molecular mechanism responsible for physiological effects mediated by this metabolite is still not fully understood. Here, we discuss evidence concerning the signalling role of melatonin and excogitate the reasons it was recruited for intercellular signalling.

### Melatonin in plant physiology

Melatonin is involved in multiple physiological processes in plants. It was implicated in abiotic stresses such as heavy metals, redox, drought, anoxia, salt, UV, heat, and cold, as well as endoplasmic reticulum (ER) stress (Arnao and Hernández-Ruiz, 2013; Bajwa et al., 2014; Li et al., 2016a; Lee and Back, 2018a; Sun et al., 2020a), and biotic stress such as the response to fungal, bacterial, and viral pathogens (Zhao et al., 2021). Melatonin was also implicated in many other physiological processes in plants, such as flowering and fruit ripening (Arnao and Hernández-Ruiz, 2020b; Byeon and Back, 2014; Mou et al., 2022), K^+^ and other ions’ homeostasis (Huang et al., 2022), nitrogen uptake and assimilation (Sun et al., 2022), and protein quality control (Lee et al., 2022). Chemically, melatonin is a potent antioxidant that can interact with ROS and reactive nitrogen species (RNS). It is estimated that one melatonin molecule can scavenge 4–10 ROS or RNS molecules. For a recent analysis of the atomic mechanism responsible for the ROS scavenging properties of melatonin, see Purushothaman et al., 2020. Crucial for the understanding of the recruitment of melatonin for intercellular signalling is the estimate that melatonin as a biomolecule originated during the transition of the earth’s atmosphere to an O_2_-enriched one (Tan et al., 2014). During that period, melatonin was mainly involved in antioxidant activities. Later, the functions of melatonin in eukaryotes diversified while evolving separately in plants and animals based on their different physiological needs.

Under many stress conditions, the application of exogenous melatonin (10–100 μM) tends to alleviate the stress (Wang et al., 2012; Zhang et al., 2012; Yin et al., 2013; Zhang et al., 2017). Moreover, under various stress conditions, the accumulation of endogenous melatonin can initiate a similar response to the exogenous melatonin treatment (Mukherjee et al., 2014). In many of these abiotic stress cases, the melatonin effect was related to a reduction of the ROS concentration and increased activity or transcript level of enzymes that catalyse ROS neutralisation or are involved in the glutathione-ascorbate cycle (Cen et al., 2020; Wang et al., 2013; Wang et al., 2012).

Many studies also reported an effect of melatonin on the RSA. In *Arabidopsis*, exogenous melatonin induced lateral root and adventitious root formation (Pelagio-Flores et al., 2012) but can also induce primary root growth at a low concentration (10–100 μM) (Bajwa et al., 2014). In many other species, such as *Mimosa pudica*, *Hypericum perforatum* (St. John’s wort), and rice, melatonin promotes root formation (Erland et al., 2018; Giridhar and Ravishankar, 2009; Liang et al., 2017b). In fact, melatonin is already known for some time as a general growth inducer in plants (Arnao and Hernández-Ruiz, 2017b; Hernández-Ruiz et al., 2005). Note, however, that, in many cases, increasing the melatonin concentration above a certain species-specific concentration level eliminates the melatonin growth induction properties or even results in growth inhibition (Chen et al., 2009; Park and Back, 2012). Several studies have shown that melatonin application results in differential expression of many genes (Zhang et al., 2013; Weeda et al., 2014; Liang et al., 2017b; Bychkov et al., 2019; Yang et al., 2021a). Particularly, exogenous melatonin in *Arabidopsis* up-regulated the expression of XTR6, a protein involved in cell-wall remodelling. This fact might explain the ability of melatonin to act as a growth stimulator (Wan et al., 2018b).

The fact that melatonin can act as a growth stimulator and the fact that it shares a common precursor (namely, tryptophan) with the plant phytohormone group auxins (one of the primary plant hormones, the most abundant member being indole-3-acetic acid [IAA]) led to the hypothesis that melatonin acts as a minute auxin (Murch and Saxena, 2002). Since then, the ground has shifted, and today melatonin is considered an important plant metabolite in its own right. Nevertheless, what is the exact relationship between melatonin and auxin is still under dispute. For example, a transcriptome comparison analysis between the pool of genes regulated by the synthetic auxin 1-naphthalene acetic acid (NAA) and that regulated by melatonin in *Arabidopsis* rosettes inferred that these two metabolites do not share a common pathway (Zia et al., 2019). By contrast, another study that compared IAA-regulated genes to melatonin-regulated genes in *Arabidopsis* found that the set of genes regulated by melatonin highly correlates with that regulated by auxin and hence concluded that melatonin works in an IAA-dependent manner (Yang et al., 2021b). Similarly, since exogenous melatonin modulates the RSA in rice and the exogenous application of melatonin resulted in differential expression of many genes associated with the auxin signalling pathway in the roots, it was claimed that melatonin controls the RSA by modulating the auxin response (Liang et al., 2017b). In parallel to the discussion concerning the relationship between melatonin and auxin, there is currently an ongoing discussion about whether melatonin should be considered a new phytohormone (Arnao and Hernández-Ruiz, 2020a) or whether it is better to conceptualise its function as a master regulator (Sun et al., 2021).

Nevertheless, alleviation of stress, changes in the RSA, or differential transcription of genes resulting from exogenous melatonin treatment does not necessarily indicate an endogenous intercellular signalling function. To be considered a bona fide intercellular signalling molecule, a molecular understanding of the signalling cascade and how melatonin confers its message over the plasma membrane must exist. Hence, we discuss below the possible molecular mechanism for the melatonin function.

### The melatonin signalling cascade

Evidence from numerous studies suggests that melatonin acts through modifying the H_2_O_2_ or nitric oxide (NO) signalling pathways (for details recent reviews, see Mukherjee, 2019; He and He, 2020 and references therein). In most of these cases, some enzymatic subunits of the plant NADPH oxidase, namely (RESPIRATORY BURST OXIDASE HOMOLOG) RBOH proteins, were implicated as necessary components in the melatonin cascade. In general, induction of the RBOH activity results in an H_2_O_2_ burst and initiation of ROS signalling. For example, in *Arabidopsis*, under salt stress, the rescue of primary root growth by melatonin depends on RBOH enzymes for downstream signalling (Chen et al., 2017). Similarly, RBOH-based signalling is also implicated in the melatonin-induced lateral root formation in alfalfa (Chen et al., 2018). Also, in tomato, RBOH-based signalling is implicated in melatonin induction of lateral root formation (Chen et al., 2019). In another recent example, the ability of melatonin to induce salinity stress tolerance was found to be dependent on AtRBOHF and AtRBOHD (Liu et al., 2020). In that case, no melatonin-induced reduction of K^+^ efflux from the root elongation zone was observed in *atrbohD* and *atrbohF* mutants. Thus, the authors suggested that melatonin influences H_2_O_2_ signalling, which results in differential protein synthesis and stress amelioration. Note, however, that H_2_O_2_ can also be produced in the cell by other means, and not only through RBOH. For example, polyamine catabolism can also result in H_2_O_2_ production. Interestingly, the production of H_2_O_2_ by polyamide oxidase is also implicated in the melatonin response cascade during the induction of lateral root in tomato (Chen et al., 2019). This fact suggests an intricate and close relationship between melatonin and H_2_O_2_. Moreover, in some of these cases, the empirical evidence suggested that melatonin acts upstream of the ROS signalling cascade.

Another key factor in the melatonin signalling cascade is NO. For example, under aluminium stress in *Arabidopsis*, melatonin alleviates root growth inhibition by repressing NO accumulation (Zhang et al., 2019). Similarly, exogenous melatonin treatment elevates the NO concentration in alkaline-stressed tomato roots and alleviates the vegetative growth of the shoots (Liu et al., 2015). In that study, the accumulation of NO results in the scavenging of stress-induced ROS (H_2_O_2_, O_2_^−^). By contrast, under drought, heat, and cold stresses in tomato, melatonin acts primarily as a NO scavenger. Reduction of the NO concentration results in denitrosylation of RBOH and subsequently increases its ROS production activity (Gong et al., 2017). In addition, in that study, melatonin treatment causes increased expression of *RBOH* itself. As a consequence, an H_2_O_2_ signalling cascade is ensued in the leaves. Another example of the crosstalk between melatonin and NO appears in adventitious root formation in tomato (Wen et al., 2016). The formation of the adventitious root is mediated by downregulating the expression of S-nitrosoglutathione reductase and, as a result, accumulation of NO in the hypocotyl. In parallel, NO also causes the accumulation of melatonin, while melatonin itself influences auxin transport and perception. Note that complex crosstalk exists between NO and H_2_O_2_ and that NO can act downstream or upstream from ROS (Zhu et al., 2019). Thus, melatonin can influence plant development and physiology by interfering with the balance between H_2_O_2_ and NO signalling.

It should be noted that not under all environmental conditions does melatonin act upstream than H_2_O_2_ and NO. In some cases, an opposite operational order was observed (Lee and Back, 2016a). These observations have led to conceptualisation where melatonin, H_2_O_2_ and NO act as a coupled-dependent triad, where each one of these signalling molecules can influence the others under some conditions (Zhao et al., 2021). In particular, it was suggested that there is a difference between the way melatonin act in biotic stress, where it promotes the production of RNS and ROS, and in their turn, ROS promote melatonin production, and abiotic stress, where melatonin reduces the amount of RNS and ROS (Zhao et al., 2021). How exactly these different responses occurs is still not fully understood, but one option that might be considered is that the difference might depend on whether melatonin function in intercellular signalling or in intracellular signalling.

Similarly, it should be noted, that not under all stress conditions, melatonin acts through ROS or NO signalling. For example, under oxidative stress in *Arabidopsis*, melatonin treatment increases the rate of autophagy in the roots independently of ROS and alleviates the stress response (Wang et al., 2015). It was suggested that this response acts as a second line of defence when ROS signalling is compromised. It is also interesting that, in animals, melatonin is implicated in a similar function of enhanced autophagy (Coto-Montes et al., 2012).

### The melatonin receptor

**Figure 6.**
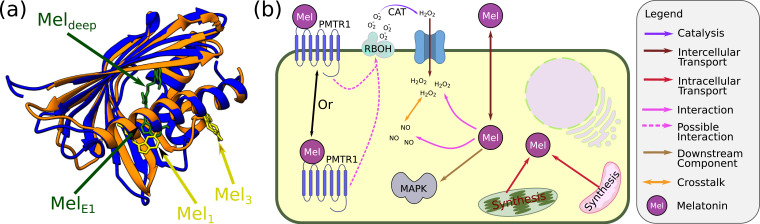
Melatonin signalling in plants. (**a**) Crystal structure of the PR-10 proteins that bind melatonin. Blue: Hyp-1 from St. John’s wort based on Protein data bank #5I8F (Sliwiak et al., 2016). Hyp-1 binds 2–3 melatonin molecules (the two with the more sound evidence are shown). Orange: LLPR-10.2B from yellow lupin with the two sites where melatonin binds the protein (based on PDB #5MXB; Sliwiak et al., 2018). (**b**) Signalling by melatonin. Due to its amphipathic properties, exogenous melatonin can shuttle between the apoplast and the symplast spaces. In both spaces, it can scavenge reactive oxygen species (ROS) and mediate ROS signalling. In the symplastic space, melatonin can also interact with reactive nitrogen species (RNS) and, in particular, nitric oxide, thereby mediating downstream NO signalling. In addition, it can interact with PMTR1, which is either a membrane or a cytoplasmic protein (still under dispute). PMTR1 can activate RBOH and initiate ROS signalling. In addition, melatonin probably works via other mechanisms that include a downstream MAPK signalling cascade.

Until very recently, no receptor or a binding partner for melatonin was identified in plants. However, as of today, at least three plant proteins are known to interact with melatonin. First, the Hyp-1 protein of St. John’s wort binds 2–3 melatonin molecules and may function in melatonin storage and sorting (Sliwiak et al., 2016). Hyp-1 belongs to a plant pathogenesis-related protein family 10 (PR-10) that is known to be involved in the storage and sorting of various phytohormones. The binding between melatonin and Hyp-1 is not very strong. Even the strongest binding pocket of Hyp-1 depends mainly on the relatively chemically weak hydrophobic interactions. Similar to Hyp-1, also the PR-10 protein from yellow lupin (LLPR10.2B) was shown to bind two melatonin molecules (Sliwiak et al., 2018). One of the melatonin-bounded molecules is buried deep inside a cavity in the protein, while a second one binds less strongly and can be displaced by the cytokinin phytohormone trans-zeatin. This cavity for the loosely bound melatonin molecule is located close to one of the melatonin-binding cavities in Hyp-1. However, Hyp-1 and LLPR10.2B bind the other melatonin molecules utterly unrelatedly (see Figure 6a for a comparison between Hyp-1 and LLPR10.2B). It was suggested that PR-10 proteins act as a reservoir for melatonin that can direct the balance between melatonin and traditional phytohormones. In this case, PR-10 proteins will act as a semi-receptors. Increasing or decreasing the concentration of melatonin can release or capture cytokinin and thus induce a response. However, the binding constant of Hyp-1 (and probably also LLPR10.2B) to different ligands is usually around 10 μM (Sliwiak et al., 2015). Since the cellular concentration of melatonin was estimated to be one to several dozen ng/ml (4.3–150 nM) (Lee et al., 2018b; Liu et al., 2019), it is not clear whether the PR-10 proteins binding to melatonin has physiological relevance.

In a different route of evidence, the exogenous application of melatonin was shown to regulate stomatal opening (Li et al., 2014a). Indeed, a third protein – COND2/PMTR1 – acting in guard cells, was shown to bind melatonin and regulate stomata opening (Wei et al., 2018). Fluorescence microscopy suggested a plasma membrane localisation and a secondary structure prediction suggested a structure with 7-transmembrane regions. Since animal G-protein-coupled receptors (GPCR) also possess a 7-transmembrane structure, it was suggested that AtPMTR1 is a GPCR melatonin receptor with a K_d_ of 0.73 nM in guard cells of *Arabidopsis*. The same study also claimed that the Gα protein GPA1 is required for the melatonin response, probably as the coupled protein of PMTR1. Indeed, exogenous melatonin was able to regulate stomata opening but failed to do so in a *pmtr1* mutant or mutants lacking in the melatonin precursor (N-acetylserotonin) synthesis enzyme (Li et al., 2020a). Moreover, both melatonin and ROS showed rhythmically controlled concentration oscillations that were associated with the stomata opening, and in the mutant lines, the level of the ROS oscillations was reduced. In guard cells, the exogenous application of melatonin induced a K^+^ efflux across the membrane and a Ca^2+^ influx (Wei et al., 2018), which depended on RBOH. In both *rbohC* and *rbohD/F* mutant, melatonin failed to module stomata opening. Hence, it was suggested that AtPMTR1 acts upstream of RBOH and Ca^2+^ signalling. Note that melatonin is an amphipathic molecule that can diffuse through the membrane, and be exported to the extracellular region without the aid of dedicated exporters. Thus, it can be suggested that, in guard cells, melatonin acts through a paracrine-like (and maybe autocrine-like) receptor-mediated mode.

Several other lines of research also suggested the involvement of PMTR1 in the melatonin-induced stress response. First, in *Arabidopsis*, it was shown that the ability of melatonin to promote osmotic-stress tolerance depended on PMTR1 (Wang et al., 2021b). In that case, the melatonin-dependent alleviation of plant growth restriction, the total amount of ROS following osmotic stress, and the expression level of several ROS processing enzymes following the stress were all AtPMTR1-dependent. Similarly, some evidence for an AtPMTR1 dependency for the melatonin alleviation of high-light stress in *Arabidopsis* also exists (Bychkov et al., 2021). Moreover, the involvement of PMTR1 in the melatonin-induced response is not restricted to *Arabidopsis*. Recent research suggested that MsPMTR1 from Alfalfa (*Medicago sativa*) is indispensable for the ability of melatonin to alleviate some detrimental consequences of salt stress (Yu et al., 2021). Similarly, in *N. benthamiana*, four homologues of AtPMTR1 were identified, and the melatonin-induced resistance against the biotic stressor *Phytophthora nicotianae* depended on at least one out of two NbPMTR1 proteins (the exact one was not identified) (Kong et al., 2021). Interestingly, however, these two NbPMTR1 proteins were the ones with the least homology to AtPMTR1 among the four identified NbPMTR1 proteins. Thus, it is possible that in different species PMTR1 evolved to respond to different environmental challenges in melatonin-dependent or -independent manners. Finally, a function for PMTR1 in the melatonin-dependent response to stress is not restricted to dicots. A function for ZmPMTR1 was also implicated in maize (*Zea mays*) defence against drought and osmotic stress (Wang et al., 2022). However, in that case, measurements suggested somewhat higher binding for melatonin (EC_50_ of 47.8 mM). Thus, if PMTR1 is indeed a melatonin receptor, this difference in binding efficiency might represent the different environmental needs of maize relative to *Arabidopsis*. Moreover, in this case, it was suggested that ROS quenching, part of the defence processes, acts downstream of the ZmPMTR1-melatonin activity. Below, we briefly discuss downstream elements from the melatonin-induced response.

However, the status of PMTR1 as a melatonin receptor in plants was also contested (Lee and Back, 2020). In that case, it was found that PMTR1 was not localised to the plasma membrane in tobacco leaves and that the ER-stressed *Arabidopsis* leaves operated through MPK3/6 independent of PMTR1. Moreover, it was claimed that PMTR1 could not be a GPCR receptor since the Gα subunit of G-proteins (that are activated by GPCR proteins in animals) are constitutively active in plants and do not need a GPCR for activation. Finally, if PMTR1 does act as a melatonin receptor in plants, it is interesting to note that it is only a very distant homologue of the animal melatonin GPCR receptors MT1 and MT2 (and GRP50) with 10–15% homology. Hence, most probably, PMTR1 and the animal melatonin receptors evolved separately from their common protein ancestor.

However, even if PR-10 proteins and/or PMTR1 do not act as melatonin receptors in plants, a simple mechanism can convey the melatonin message over the plasma membrane. Again, this mechanism is dependent on the amphipathic nature of melatonin that enables it to diffuse through the plasma membrane and interact directly with components of its signalling cascade (e.g., H_2_O_2_ and NO, as was discussed).

### Melatonin cascade downstream from ROS, NO, and RNS

Several studies connected the melatonin signalling cascade to the MAPK signalling cascade (Lee and Back, 2016b; Lee and Back, 2018a). In particular, it was found that during biotic stress in *Arabidopsis*, melatonin acts through two MPEKKK, namely MAPKKK3 and OXI1 that act upstream of a MAPKK4/5/7/9-MAPK3/6 module (Lee and Back, 2016a). In another recent example, it was suggested that melatonin is involved in chloroplast protein quality control and that this involvement is mediated by two pathways, a MPK3/6-dependent one and an MPK3/6-independent one (Lee and Back, 2021). However, in that case, it is not clear whether melatonin acts as an intercellular or as intracellular signalling molecule or whether PMTR1 is involved in the process. Finally, in *Panax notoginseng*, melatonin induced the expression of PnMKK4/5 and PnMPK3/6 as well as PnMPK3/6 phosphorylation in the context of the plant innate immune and stomatal closure response to biotic stress (Yang et al., 2021c). Interestingly, in that case, in a *ptmr1 Arabidopsis* mutant, melatonin failed to induce the stomatal closure and the innate immune response. Moreover, in a *gpa1 Arabidopsis* mutant, melatonin failed to increase bacterial resistance but not the phosphorylation of MPK3/6. Hence, it was suggested that two independent pathways act downstream of melatonin and PTMR1, one through MPK3/6 and the second through GPA1 (Yang et al., 2021c). Note that downstream transcription factors were also implicated in the melatonin response (Murata et al., 2015; Shi et al., 2015a; Shi et al., 2015b; Shi et al., 2018), and for at least one of these, a link to the MAPK cascade might also be implicated (Liu et al., 2013).

Even downstream from the MAPK cascade or independent of it, melatonin treatment affects several phytohormones (Arnao and Hernández-Ruiz, 2017a), such as ethylene (Arnao and Hernández-Ruiz, 2007), cytokinin (Zhang et al., 2017), brassinosteroid (Hwang and Back, 2018), and salicylic acid (Lee et al., 2015) or gibberellic acid (through the control by brassinosteroid) (Hwang and Back, 2019). Some stress was directed to the connection between melatonin and ABA (Li et al., 2014a), though interestingly, melatonin acts both through ABA-dependent and ABA-independent pathways (Fu et al., 2017). However, as was discussed above, most of the discussion concerning the influence of melatonin on phytohormones was directed to the possible synergetic crosstalk of melatonin with auxin (Sarropoulou et al., 2011; Shi et al., 2014; Wang et al., 2016; Ren et al., 2019). Note, however, that some phytohormones, such as ABA and ethylene, also act upstream of melatonin (Arnao and Hernández-Ruiz, 2013).

### Long-distance melatonin effects

Under local cold stress, melatonin may function as a long-distance intercellular signalling molecule in *Citrullus lanatus* (watermelon) (Li et al., 2017). Under these conditions, melatonin induces responses at far-away organ sites. Specifically, cold stress sensed by the shoot with melatonin treatment results in a ROS-based reaction and differential gene expression in the roots and vice versa. An increased exudation of melatonin into the xylem (and possibly the phloem) combined with passive transport probably mediates this effect. Similarly, the application of melatonin in the soil resulted in foliar accumulation of melatonin in the leaves of maize (*Z. mays*) (Yoon et al., 2019). The accumulation did not reach saturation but was blocked by the closure of the stomata. This effect suggests that it was transported through the xylem. However, visualising melatonin conjugated quantum-dots in St. John’s wort under non-stress conditions suggested that the melatonin is excluded from vascular bundles and is concentrated in the endodermal and pericycle cells (Erland et al., 2019). Naturally, the in vivo melatonin transport mechanism might be hindered or biased as a result of the conjugation to the bulky quantum dots. There was also a suggestion that melatonin can act as a long-distance intercellular signalling molecule in the form of NO-melatonin conjugate (Mukherjee, 2019). The empirical evidence for such a signalling mode is currently lacking. Thus, though some evidence suggests endocrine signalling of melatonin in plants, it is not clear whether this is the case, and if it is, what is the mechanism.

### Melatonin signalling: Summary

Collectively, the evidence discussed shows that melatonin participates in important signalling cascades in plants. These signalling cascades influence various physiological processes that are not directly related to the synthesis or degradation of melatonin itself. Hence, melatonin is not only a simple metabolite in plants (see Figure 6b). Particularly, melatonin probably acts through secondary ROS (and especially H_2_O_2_) signalling. This mode of action might depend on putative receptors (such as PMTR1) and RBOH. Subsequently, secondary ROS signalling and its crosstalk with the NO signalling cascades enable melatonin signalling to crosstalk with various other phytohormone signalling pathways. The second route of melatonin intercellular signalling might be receptor-independent. Since melatonin is an amphipathic molecule, it can directly enter the cell (or be transferred between different organelles) and directly scavenge RNS, ROS, and NO. Consequently, ROS and NO signalling are modified. In both cases, the result is a differential control over the transcription of various genes. These modes of action are probably primarily activated under various stress conditions. Alternatively, melatonin may act through ROS/NO-independent route, which may also depend on specific stress conditions. The main modes of action of melatonin are probably paracrine-like and autocrine-like ones, though an endocrine-like mode was also suggested.

So why was melatonin recruited for intercellular signalling in plants? Unlike glutamate and GABA, melatonin is not a key molecule in the metabolic network. However, two properties make melatonin an excellent candidate to act as an intercellular signalling molecule: first, its function as a ROS antioxidant, and second, its ability to pass the plasma membrane passively. The ability of melatonin to act as an ROS scavenger that was implicated during its initial recruitment for intercellular signalling in plants is still implicated in its intricate crosstalk with H_2_O_2_ and NO today. Naturally, dedicated receptors can enhance the specificity and accuracy of information conveyed by melatonin. Indeed, in animals, the melatonin receptors are highly studied. In contrast, the discussion concerning putative receptors is ongoing in plants. Nevertheless, the ability of melatonin to crosstalk with other signalling molecules, particularly its ROS scavenging properties, enables it to interact with necessary additional machinery needed for signalling. Thus, the probable evolutionary scenario in plants and animals starts with melatonin participating in ROS scavenging. Subsequently, both animals and plants recruited this molecule for intercellular signalling due to its amphipathic nature. Finally, dedicated receptors and other necessary machinery evolved separately in plants and animals.

## Serotonin, dopamine, and acetylcholine

Though serotonin, dopamine, and acetylcholine exist in plants and were implicated in plant physiology, to the best of our understanding, molecules from this triad do not act as intercellular signalling molecules. Here, we briefly discuss the reasons for our conclusion.

### Serotonin

Over the years, it has been suggested multiple times that serotonin may act as an intercellular signalling molecule in plants (Abbasi et al., 2020; Ramakrishna et al., 2011; Mukherjee, 2020). For example, recently it was hypothesised that serotonin synthesised in the vasculature controlled the suberisation of endodermal/exodermal cells via a specialised transport and receptor system that is yet not have been identified (Lu et al., 2022). Based on the current evidence, our view, however, is different. Indeed, exogenous serotonin can act as a growth simulator in plants especially concerning the RSA (Pelagio-Flores et al., 2011; Pelagio-Flores et al., 2016; Wan et al., 2018b). In addition, it serves as an important metabolite during biotic defence (Ishihara et al., 2008; Dharmawardhana et al., 2013; Hayashi et al., 2016; Lu et al., 2018), abiotic defence (He et al., 2021; Mukherjee et al., 2014; Shukla et al., 2021; Lu et al., 2022), senescence (Kang et al., 2009), and can act as in ROS scavenging (Bajwa et al., 2015). However, as of today, no serotonin receptor has been identified in plants. There is also no knowledge of a dedicated importer, exporter, or another mechanism to cross the plasma membrane. Since, in contrast to melatonin, serotonin is less amphipathic and does not translocate across membranes easily (Yu et al., 2016), its distribution between the apoplastic and the symplastic spaces is probably quite restricted. Also, to the best of our knowledge, no report was made concerning the transport of serotonin through the plasmodesmata. Note that serotonin can diffuse through the membrane but very slowly. Hence, its ability to act as an intercellular messenger molecule is probably also limited. Exogenous serotonin probably works through ROS scavenging in the apoplast, while endogenous serotonin probably acts through a parallel mechanism in the symplastic space. However, it is hard to conceive a mechanism that transduces the effects of serotonin in the apoplastic space to the symplastic space and vice versa that is not primarily dependent on other intercellular signalling molecules. Since serotonin does not fulfil our criteria (ii) from above, we conclude that, by contrast to animals, as of today, serotonin cannot be considered an intercellular signalling molecule in plants despite its widespread documented physiological effects. Naturally, since serotonin can slowly pass membranes, it is hard to decide whether there are not some circumstances where plants use serotonin for intercellular communication. However, positive evidence for this effect is missing. Thus, although serotonin might have been recruited for intercellular signalling in plants due to its physiological function, this scenario is unlikely. Note, however, that this conclusion also does not contradict the possibility of serotonin acting as a secondary messenger in signalling pathways or as an intracellular signalling molecule.

### Dopamine

Similar to serotonin, also dopamine was implicated in plant physiology (Kanazawa and Sakakibara, 2000; Kulma and Szopa, 2007). In particular, there is evidence for the action of dopamine in ROS balancing (Guidotti et al., 2013; Gomes et al., 2014; Stefi et al., 2019), abiotic stress alleviation (Abdelkader et al., 2012; Li et al., 2014b; Liang et al., 2017a; Liang et al., 2018), and even biotic stress alleviation (Swiedrych et al., 2004). Yet, one of the problems with ascribing dopamine with an intercellular signalling role in higher plants is the lack of evidence for the existence of a receptor, though some fluorescent antagonists of dopamine bind to plant cell membranes (Roshchina, 2016). By contrast, in green algae, some evidence suggests a possible existence of a functional dopamine receptor. In particular, in the green algae *Chara corallina*, dopamine and its antagonists modulate the ion current through a membrane patch (Kataev et al., 2019). Similarly, a screen of small molecules that influence flagella length and motility in the green algae *Chlamydomonas reinhardtii* identified molecules known to interact with the family of dopamine-binding GPCRs in vertebrates (Avasthi et al., 2012). In addition, catecholamines are, in general, relatively impermeable to membranes (Yu et al., 2016). Hence, there are limited possibilities for communication between the apoplastic and the symplastic spaces through passive diffusion. Thus, since in higher plants (i) no dopamine receptor, (ii) no way to communicate between the apoplastic and the symplastic spaces, and (iii) no transport through plasmodesmata were identified, dopamine cannot be considered as an intercellular signalling molecule in plants based on the current evidence. Most probably, similar to serotonin, endogenous dopamine primarily protects the cell from ROS but may also interfere with ROS signalling. Exogenous dopamine probably influences plants via a similar mechanism in the apoplastic space.

### Acetylcholine

In animals, the acetylcholine (ACh) system is usually defined together with its receptor (AchR), the ACh catabolisiser acetylcholinesterase (AChE), and the synthesiser of ACh, choline acetyltransferase (ChAT). Of these three proteins/enzymes, the best-identified example in plants is AChE (Sagane et al., 2005). However, plant AChE is not a homologue of animal AChE. Fundamental differences exist between the chemical characteristics of these animal and plant AChE enzymes (Sagane et al., 2005). Thus, not every physiological effect that was attributed in the literature to AChE can immediately be interpreted as related to ACh. ChAT activity was also detected in plants (Kawashima et al., 2007). However, this enzyme is probably also not a homologue of the animal one. By contrast, the identification of AChR is restricted to unique cases (Yoshida et al., 2019).

ACh itself was suggested to influence several physiological processes (e.g. development, flowering, and more) (Erland and Saxena, 2019). It was also suggested that ACh could participate in abiotic stress alleviation (Qin et al., 2020) or pollen-tube growth (Tezuka et al., 2007). Also, some studies suggested a possible role in adventitious or lateral root formation (Bamel et al., 2007; Sugiyama and Tezuka, 2011; Murata et al., 2015). In the past, plant ACh attracted some attention and was implicated in several physiological processes. However, little evidence based on modern molecular biology techniques suggests a signalling function for ACh in plants (Bamel and Gupta, 2018). Several years ago, one study suggested that ACh can act synergistically with auxin (but not alone) to promote the expression of expansin, a protein that mediates the loosening of the cell wall hydrogen bonds and hence cell growth (Di Sansebastiano et al., 2014). Still, there is a lack of modern evidence or a mechanism for an ACh signalling function in plants. Similarly, we are not aware of any mechanism in plants that will permit conveying ACh putative message over the plasma membrane. Thus, we conclude that, as of today, one cannot attribute an intracellular signalling function to acetylcholine in plants.

### Outstanding questions

1. Does glutamate act as a GLR-dependent intercellular signalling molecule in processes other than wounding?
2. Does glutamate-dependent restructuring of the RSA represent a glutamate-dependent intercellular signalling event?
3. What is the exact relationship between GABA acting as a signalling molecule and GABA working in metabolism?
4. Does the action of GABA on ALMT channels result in differential expression of proteins or only in transport of ions to and from the cytoplasm?
5. What is the exact repertoire of agonists and antagonists for different ALMT channels?
6. What is the exact role of COND2/PMTR1 in melatonin signalling? Are there other receptors for melatonin in plants?
7. Which signalling mechanisms of the glutamate-GABA-melatonin triad operated in plants’ and animals’ last common ancestor, and which evolved separately?
8. Do receptors or other manners to cross the membrane for serotonin and dopamine exist in plants? If they exist, that may suggest that these metabolites also act as intercellular signalling molecules in plants.
9. What evolutionary forces cause the recruitment of molecules acting in metabolism and ROS scavenging to become intercellular signalling molecules?
10. What are the evolutionary forces that cause certain molecules to become intercellular signalling molecules in multicellular organisms?

### Conclusions

Today, there is evidence that glutamate, GABA, and melatonin (aka the first triad) act as signalling molecules in plants. Mostly, this evidence also suggests an intercellular signalling function. We contrast this triad with three other well-studied animal intercellular signalling molecules that function in plants. These are serotonin, dopamine, and acetylcholine. We call these molecules the second triad. For the second triad, no evidence or no identified mechanism exists that shows they act as intercellular signalling molecules in plants. This contrast is important since it was claimed in some publications that also molecules from the second triad act as signalling molecules in plants (Akula and Mukherjee, 2020; Erland and Saxena, 2019). We summarise the evidence (or lack of evidence) for these molecules in intercellular signalling in plants in Table 1, and their effects on the RSA as an example of a specific physiological mechanism that is not necessarily intercellular signalling in Table 2.

**Table 1.** Main indication of the first triad function in plant signalling and the second triad function in plant physiology. Yellow background: molecules from the first triad that act as plant signalling (and particularly intercellular) molecules and the best examples for their participation in signalling processes. Green background: molecules from the second triad where there is currently a lack of evidence of their participation in intercellular signalling in plants and examples of processes where they do participate. References related to the figure are glutamate (Kwaaitaal et al., 2011; Li et al., 2019; López-Bucio et al., 2018; Mousavi et al., 2013; Nguyen et al., 2018; Toyota et al., 2018; Walch-Liu et al., 2006; Wang et al., 2019a; Yoshida et al., 2016); GABA (AlQuraan, 2015; Guo et al., 2020; Li et al., 2016a; Malekzadeh et al., 2013; Mustroph et al., 2014; Palanivelu et al., 2003; Ramesh et al., 2015; Seifikalhor et al., 2020; Wang et al., 2014; Wang et al., 2017a; Xu et al., 2021a); melatonin (Chen et al., 2019; Chen et al., 2018; Chen et al., 2017; Erland et al., 2018; Giridhar and Ravishankar, 2009; Li et al., 2014a; Liang et al., 2017b; Pelagio-Flores et al., 2012; Tan et al., 2014; Wei et al., 2018; Zhang et al., 2019; Zhu et al., 2019); serotonin (He et al., 2021; Kang et al., 2009; Mukherjee et al., 2014; Pelagio-Flores et al., 2016; Pelagio-Flores et al., 2011; Shukla et al., 2021; Wan et al., 2018a ; Wan et al., 2018b); dopamine (Dai et al., 1993; Gomes et al., 2014; Guidotti et al., 2013; Protacio et al., 1992; Stefi et al., 2019); and acetylcholine (Bamel et al., 2015; Bamel et al., 2007; Bamel and Gupta, 2018; Di Sansebastiano et al., 2014; Erland and Saxena, 2019; Qin et al., 2020; Tezuka et al., 2007). Note that the reference list is not exhaustive. For full references, see the main text. Also, for full details of the effects related to each molecule, see the full text.

Molecule		Effect	
		Processes indicative of signalling/participation in physiology	Putative receptor
**Signalling**	**Glutamate**	Implicated in the formation of calcium waves and systemic electric potential propagation following root-nematodes attack and leaf wounding. Implicated in restructuring the root system architecture. Initial evidence suggests a function in guard cells. Participates in biotic and abiotic defence mechanisms.	**GRL3.3** **GRL3.6** **SlGLR3.5**
	**GABA**	Participates in the regulation of stomatal-aperture. Implicated in the regulation of Al^3+^ stress by controlling malate exudation. Some evidence suggests a role in pollen tube guidance. Participates in biotic and abiotic defence mechanisms.	**ALMT1** **ALMT9** **ALMT12**
	**Melatonin**	Participates in the modulation of NO and H_2_O_2_ signalling. Participates in the regulation of stomatal-aperture. Implicated in restructuring the root system architecture. Participates in balancing reactive oxygen and nitrogen species in particular during stress.	**PMTR1** **RD-10**
**Non-signalling**	**Serotonin**	Implicated in restructuring the root system architecture. Participates in protection against biotic and abiotic stress. Implicated in senescence mechanism.	–
	**Dopamine**	Provides protection against biotic stress by regulating reactive oxygen species. Influence the auxin-cytokinin-ethylene phytohormone balance.	–
	**Acetylcholine**	May participate in processes related to development and flowering. May also distinguish between self-pollination and cross-pollination. May act with Auxin in modulating cell wall strength. Can increase salt tolerance. May influence the root system architecture.	–

**Table 2.** Summary of the effects of different molecules discussed in this article on the plant root system. For the exogenous glutamate effect, see Figure 2 in Walch-Liu et al., 2006. For the exogenous GABA effect, see Figure 1 in Barbosa et al., 2010, Figure 2 in Xie et al., 2020, and Ramesh et al., 2015. For exogenous treatment with melatonin, see Figure 2 and Figure 5 in Pelagio-Flores et al., 2012, Figure 1 in Bajwa et al., 2014, Figure 1 in Liang et al., 2017b, and Erland et al., 2018. For the exogenous serotonin effect, see Figure 2 in Pelagio-Flores et al., 2011, Figure 1 in Pelagio-Flores et al., 2016, Figure 1 in Wan et al., 2018b, and Figure 1 in Wan et al., 2018a. For exogenous dopamine effect, see Figure 3 in Liang et al., 2017a. For the exogenous acetylcholine effect, see Figure 2 in Sugiyama and Tezuka, 2011, Figures 5 and 6 in Murata et al., 2015, and Figure 1 in Bamel et al., 2007. Note that the reference list is not exhaustive. For full references, see the main text. Conc: concentration. Yellow background: metabolites that are considered to be intercellular signalling molecules by our criterion. Green background: metabolites that are not considered to be intercellular signalling molecules by our criterion.

Molecule	Effect
	Effect on the root system architecture
**Glutamate**	Inhibits primary root growth Increases lateral root density Induces the formation of long lateral roots near the root tip Reduces distance to the first root hair from the root tip
**GABA**	Inhibits primary root growth Delays adventitious root formation Alleviates Al^3+^-inhibition of root growth
**Melatonin**	Induces primary root growth at a low concentration Promotes lateral root formation Promotes primary root growth at low concentration Induces adventitious roots
**Serotonin**	Induces the lateral root development at a low concentration Inhibits the primary root growth and induces shoot-derived adventitious root formation at high concentration
**Dopamine**	Increases ion micronutrient (and macronutrient) absorption from the soil, thereby alleviates salt stress
**Acetylcholine**	Promotes lateral root growth from radish etiolated seedlings Inhibits the primary root growth in *Arabidopsis* Promotes the formation of roots from tomato leaf explants

What type of biological insight can be gained from the current survey? We divide such insight into two levels – the empirical case-specific one and the general principle hypothetical one. On the case-specific level, one can see that in many cases the plant system essential for the function of the signalling triad is not homologous to the one that acts in animals. When receptors were identified in plants, in many cases, they are not related to the animal ones, for example, the melatonin receptor, ALMT. In other cases, there can be a close homology between receptors of animal signalling molecules and the plant ones, for example, the GLRs. However, in these cases, the plant receptors do not show specificity toward the corresponding animal signalling molecule, for example, the broad agonist repertoire of the GLRs. Similarly, enzymes needed for synthesising or breaking down of the intercellular signalling molecules in plants are not necessarily homologues of the animal ones, for example, the different pathways for melatonin synthesis in plants and animals. In fact, the last conclusion is also correct for the second triad (see acetylcholine esterase [AChE] enzyme). However, in some cases, it seems that, even if the plant protein (receptor or enzyme) is not a homologue of the animal one, the active site can be a homologue of the animal protein’s active site. In these cases, only a short peptide from the plant and animal protein sequences shares a homology. These cases also caution when using animal agonists or antagonists of signalling molecules in plant research. To stress this point, antagonists and agonists that act in animals often interact with active sites of plant proteins. Nevertheless, this does not necessarily suggest that the protein machinery is conserved between animals and plants, even if the animal agonist or antagonist does have a physiological phenotypic outcome in plants.

On the more general principle level, we ask ourselves why this triad acquired signalling function, particularly intercellular signalling, in animals and plants. One option is that non-signalling physiological functions in the last common ancestor of Plantae and Animalia in metabolism (GABA and glutamate), pH regulation (GABA), and ROS quenching (melatonin) are responsible for their recruitment for their intercellular signalling functions separately in animals and plants. The second option is that similar biological forces were already in action in the last common ancestor of plants and animals. The first scenario is probably the case for GABA and melatonin. In that case, the evolving organisms used whatever was in their hands and built around it the necessary machinery according to their needs. By contrast, glutamate was probably recruited for cell-to-cell communication in plants’ and animals’ unicellular ancestor. Moreover, the machinery needed for cell-to-cell communication existed before the separation between animals and plants. However, the intercellular signalling machinery in each kingdom was shaped according to its specific evolutionary path. Nevertheless, in all three cases, a pivotal role in metabolism and/or ROS balancing makes a molecule a good candidate to become an intercellular signalling molecule. In other words – if organisms have a specific molecule acting centrally in metabolism or reactive ion species balancing, there will be an evolutionary tendency to recruit it for intercellular signalling and evolve the necessary machinery to convey its message. This fact is probably also why this triad was recruited for intercellular signalling in animals, particularly in the CNS. Nevertheless, from the fact that all molecules from the first triad operate in plants mainly under specific biological conditions and do not act similarly to general plant hormones (such as auxin), one can hypothesise that the selection of multi-function signalling molecules is a complex evolutionary process.

But one can continue and ask if a pivotal role in metabolism and/or ROS balancing is a sufficient condition for the recruitment to intercellular signalling. Naturally, the answer is negative as many plant and animal metabolites with a pivotal role in metabolism and/or ROS balancing did not evolve to become bona fide intercellular signalling molecules. So, what else is required? Evidence from the evolutionary history of some plant phytohormone signalling, besides the glutamate/GABA/melatonin triad, might shed additional light on the evolutionary recruitment process concerning this triad. For example, it was suggested that the major plant phytohormone auxin originally acted only as an important metabolite and that the necessity to overcome its toxicity led to a mechanism for its secretion (Vosolsobě et al., 2020). Thus, it was suggested that the secretion process was the driving force for its intercellular functions. In addition, another source of auxin might have been fungi and bacteria in the environment during the evolution of land plants. Hence, the interaction of land plants with their microbiota and their internal metabolic needs together might have also promoted the adaptation of auxin to intercellular signalling. A similar recent suggestion, based on extensive phylogenetic evidence, also emphasised the role of fungal and prokaryotic auxin production in the evolutionary pathway that led to auxin’s adaptation for intercellular signalling (Carrillo-Carrasco et al., 2023). In that case, the suggested evolutionary scenario is based on the prominent role of auxin in the phycosphere, plus the adaptation of a previously available response pathway that originally was not associated with auxin. These two factors were the driving source for this evolutionary process.

Also other phytohormones could have origins from the plant microbiome. In the case of ABA, it was also suggested that an ancient response independent of ABA was later recruited to interact with ABA in the context of the stress response (Sun et al., 2019; Sun et al., 2020b). However, this adaptation would not have occurred if ABA would have not already being produced by the plants in the context of other, still unknown, functions. It is also known that ABA functions in plant–microbe interactions (Shahzad et al., 2017) and in plant–arbuscular mycorrhiza symbioses (Bedini et al., 2018). Furthermore, other evidence shows that bacteria produce another phytohormone, cytokinin, that some components of the cytokinin were identified in prokaryotes and fungi (Rashotte, 2021), and that bacteria make use of this phytohormone for their interactions with plants (Giron et al., 2013). This evidence might also suggest an inter-organism origin for the intercellular signalling function of ABA and cytokinin in plants.

Is there any evidence that suggests that the glutamate/GABA/melatonin triad has some function in the environment or in microbiota–plant interaction that led to their adoption for intercellular signalling? As for melatonin, its ability to cross the plasma membrane unassisted might have been the driving force. Moreover, interestingly, it was recently found that endophytic bacteria produce melatonin and use it for communication with plant cells (Jiao et al., 2016). Naturally, this melatonin production by microorganisms might have been a later adaptation by the microorganisms to their specific environment. But such communication might also represent an ancient process from the early days of land plants. Thus, also in the case of melatonin, as is probably in the case of auxin and cytokinin, microorganisms in the environment of ancient plants might have contributed to its current intracellular signalling functions. We believe that such a hypothesis deserves further research. As for GABA and glutamate, while we are not aware of any report that microorganisms use them for communication with plants, it is known that these two metabolites are among the most abundant metabolites in many soils (Warren, 2017). In that case, a study of microbial extract from soils from multiple altitudes suggested that glutamic acid was among the most abundant proteinous amino acids in the soil, while GABA could amount to more than 50% of the non-proteinous amino acids in these soil samples. Moreover, as was discussed above, at least for glutamate, it was suggested that it plays an important role in plant–environment interactions. Hence, it may have been the case that, besides the role of these two metabolites in metabolism, plant–microbe or plant–environment interactions were also necessary for their adaptation to intercellular signalling in plants. We believe that also this hypothesis deserves further research.

Finally, we can also speculate that similar evolutionary forces (i.e. pivotal role in metabolism and/or ROS balancing plus microbe interactions) might also have been vital for the adaptation of melatonin, GABA, and glutamate for intercellular signalling in animals and in particular in the CNS. Indeed, it is known that gut microbes produce and consume GABA or can use glutamate to produce GABA in the context of their gut–brain interaction (Barrett et al., 2012; Strandwitz et al., 2019). Similarly, the gut microbes and melatonin are involved in intricate bidirectional interrelationships (Iesanu et al., 2022).

We hope that this review will stimulate further research on intercellular signalling in animals and plants, but also contribute to a better understanding of plant physiology per se. Nevertheless, several research directions should probably be taken to push forward our understanding concerning the evolutionary forces responsible for the recruitment of molecules for intercellular signalling. First, animal-centred and plant-centred scientists should be better informed about parallels and differences in intercellular signalling processes in these two kingdoms. For example, animal-centred scientists should be more aware of signalling processes in animals associated with plant hormones such as salicylic acid and oxylipins (the precursors of jasmonate) and with more general signalling molecules such as NO and H_2_O_2_. Similarly, plant-centred scientists should use modern molecular biology techniques to fully understand the mode of action of dopamine-acetylcholine-serotonin in plants and continue to develop our understanding of glutamate-GABA-melatonin signalling in plants.

Second, a common language, or at least a recognition of the reference to similar processes by scientists concentrating on the different kingdoms, should be set in place. For example, plant and animal-centred scientists often use different terms to denote similar signalling processes. We followed this line using animal-centred language to analyse the plant case.

Third, research about signalling will have to depart from the model organism approach, and a much larger emphasis should be placed on studying non-model organisms, especially these that are related to the last common ancestor of land plants or these that are related to the last common ancestor of animals. This approach includes cell-to-cell signalling in unicellular protozoa, intercellular signalling in red algae (as an outgroup example of green plants), and intercellular signalling in slime moulds (as an outgroup example for animals and fungi). In addition, scientists should apply modern genetic, transcriptomics, and metabolomics experimental tools to understand how cell-to-cell and simple intercellular signalling function in these cases.

However, maybe most importantly, plant- and animal-centred scientists should come together while maintaining their separate interests and field of study and learn from each other’s examples. This cooperation can be realised, for example, by organising joint conferences or increasing visibility and awareness of cross-kingdom similarities and differences. We believe that this recommendation is especially relevant for animal-centred scientists as plant-centred scientists are usually more aware of developments and concepts from the animal world. Such cross-disciplinary fertilisation can help in advancing the evolutionary theory of intercellular signalling. We hope that this review is a step in this direction.
